# Highly efficient and stable binary and ternary organic solar cells using polymerized nonfused ring electron acceptors

**DOI:** 10.1093/nsr/nwae258

**Published:** 2024-08-12

**Authors:** Xiaodong Wang, Nan Wei, Ya-nan Chen, Guangliu Ran, Andong Zhang, Hao Lu, Zhengdong Wei, Yahui Liu, Wenkai Zhang, Zhishan Bo

**Affiliations:** College of Textiles & Clothing, State Key Laboratory of Bio-fibers and Eco-textiles, Qingdao University, Qingdao 266071, China; Beijing Key Laboratory of Energy Conversion and Storage Materials, College of Chemistry, Beijing Normal University, Beijing 100875, China; College of Textiles & Clothing, State Key Laboratory of Bio-fibers and Eco-textiles, Qingdao University, Qingdao 266071, China; School of Physics and Astronomy, Applied Optics Beijing Area Major Laboratory, Center for Advanced Quantum Studies, Beijing Normal University, Beijing 100875, China; College of Textiles & Clothing, State Key Laboratory of Bio-fibers and Eco-textiles, Qingdao University, Qingdao 266071, China; College of Textiles & Clothing, State Key Laboratory of Bio-fibers and Eco-textiles, Qingdao University, Qingdao 266071, China; College of Materials Science and Engineering, Qingdao University, Qingdao 266071, China; College of Materials Science and Engineering, Qingdao University, Qingdao 266071, China; College of Textiles & Clothing, State Key Laboratory of Bio-fibers and Eco-textiles, Qingdao University, Qingdao 266071, China; School of Physics and Astronomy, Applied Optics Beijing Area Major Laboratory, Center for Advanced Quantum Studies, Beijing Normal University, Beijing 100875, China; College of Textiles & Clothing, State Key Laboratory of Bio-fibers and Eco-textiles, Qingdao University, Qingdao 266071, China; Beijing Key Laboratory of Energy Conversion and Storage Materials, College of Chemistry, Beijing Normal University, Beijing 100875, China

**Keywords:** organic solar cells, all-polymer organic solar cells, ternary blend strategy, nonfused ring electron acceptors

## Abstract

This study reports the successful design and synthesis of two novel polymerized nonfused ring electron acceptors, **P-2BTh** and **P-2BTh-F**, derived from the high-performance nonfused ring electron acceptor, 2BTh-2F. Prepared via Stille polymerization, these polymers feature thiophene and fluorinated thiophene as π-bridge units. Notably, **P-2BTh-F**, with difluorothiophene as the π-bridge, exhibits a more planar backbone and red-shifted absorption spectrum compared with **P-2BTh**. When employed in organic solar cells (OSCs) with PBDB-T as the donor material, **P-2BTh-F**-based devices demonstrate an outstanding power conversion efficiency (PCE) of over 11%, exceeding the 8.7% achieved by **P-2BTh**-based devices. Furthermore, all-polymer solar cells utilizing PBDB-T:**P-2BTh-F** exhibit superior storage stability. Additionally, **P-2BTh-F** was explored as a functional additive in a high-performance binary system, enhancing stability while maintaining comparable PCE (19.45%). This strategy offers a cost-effective approach for fabricating highly efficient and stable binary and ternary organic solar cells, opening new horizons for cost-effective and durable solar cell development.

## Teaser

This study introduces a novel approach to solar cell technology by synthesizing high-performance nonfused ring electron acceptors for all-polymer solar cells, which bolster their stability and cost-effectiveness.

## INTRODUCTION

In recent years, organic solar cells (OSCs) have garnered significant attention due to their numerous advantages, including exceptional mechanical flexibility, lightness, and semi-transparency [1–10]. The concept of bulk heterojunctions (BHJs), consisting of p-type and n-type molecules, has been widely utilized in OSCs. This has led to remarkable progress in device performance, with power conversion efficiency (PCE) exceeding 19% [11–16]. As we look towards future applications, the stability of OSCs has become increasingly important [17]. The active layer, responsible for absorbing and converting sunlight, is a critical component of OSCs. The morphological stability of the BHJ active layer plays a crucial role in device performance. According to previous research, the active layer in its metastable state often exhibits instability after prolonged storage or exposure to light and thermal aging [18–20]. For active layers composed of polymer donors and small molecular acceptors, their instability is likely due to the tendency of small molecular acceptors to aggregate into larger domains and develop numerous structural defects over time [21,22]. To address these issues, the polymerization of small-molecule acceptors has received increasing attention as a means of suppressing the migration of small-molecule acceptor materials and enhancing morphological stability [23–26]. However, a significant challenge lies in the fact that polymerized small-molecule acceptors primarily rely on Y-series acceptor derivatives, which involve complex synthesis routes and relatively high costs [27,28].

In recent times, researchers have proposed an innovative concept of nonfused ring electron acceptors (NFREAs) to reduce the cost of acceptor materials in OSCs [29–33]. By employing strategies such as intramolecular noncovalent bond interactions and steric hindrance units, researchers have successfully developed and designed a series of high-efficiency and low-cost nonfused ring acceptor materials [34–36]. Currently, the efficiency of organic photovoltaic devices based on the most advanced NFREAs has surpassed 17%, indicating immense potential for future applications [31,36]. Given the significance of cost and stability, the polymerization of NFREAs emerges as a potentially effective approach to address the cost and stability challenges of OSCs. However, there is a notable scarcity of reported research in this particular area. Therefore, it is imperative to undertake a thorough investigation of the intricate relationship between molecular structure and performance, with the ultimate aim of developing high-efficiency polymerized NFREAs.

In this study, we successfully designed and synthesized two novel polymerized NFREAs, **P-2BTh** and **P-2BTh-F**, leveraging the high-performance NFREA, 2BTh-2F, as our starting point. These polymers, **P-2BTh** and **P-2BTh-F**, can be conveniently prepared via Stille polymerization, where thiophene and fluorinated thiophene serve as the π-bridge units. Notably, **P-2BTh-F**, featuring 3,4-difluorothiophene as the π-bridge, exhibits a more planar backbone and a red-shifted absorption spectrum compared to **P-2BTh**. When using PBDB-T as the donor material in OSCs, devices based on **P-2BTh-F** exhibit an outstanding PCE of over 11%, surpassing the 8.7% achieved by **P-2BTh**-based devices. Furthermore, all-polymer solar cells utilizing PBDB-T:**P-2BTh-F** demonstrate superior storage stability. In addition, we have tested the role of **P-2BTh-F** as a functional additive in high-performance binary systems composed of polymer donors and small-molecule acceptors (PBDB-T:Y18-1F or D18:L8-BO). Remarkably, the ternary devices exhibited comparable PCE while significantly enhancing stability. By considering both stability and cost, our strategy has successfully crafted highly efficient and remarkably stable binary and ternary OSCs using polymerized NFREAs. This advancement paves the way for the development of cost-effective and long-lasting solar cells.

## RESULTS AND DISCUSSION

**Scheme 1.**
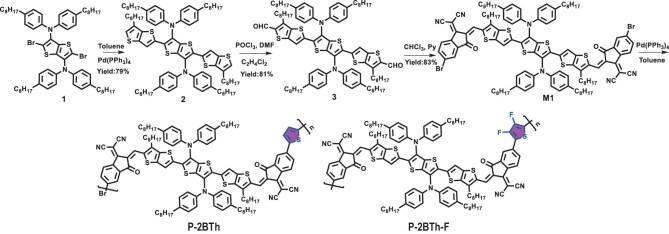
Synthesis of **P-2BTh** and **P-2BTh-F**.

The chemical structures and synthetic route of **P-2BTh** and **P-2BTh-F** are shown in Scheme 1. The starting material (compound 1) can be synthesized according to previous literature [37]. Subsequently, compound 2 can be obtained by the Stille coupling reaction in a yield of 79%, and then converted to compound 3 by the Vilsmeier–Haack reaction. The monomer M1 is synthesized by Knoevenagel condensation between compound 3 and 2-(5-bromo-3-oxo-2,3-dihydro-1H-inden-1-ylidene)malononitrile. The polymers **P-2BTh** and **P-2BTh-F** are synthesized separately through Stille coupling polycondensation reactions between monomer M1 and 2,5-bis(trimethylstannyl)thiophene (M2), and between monomer M1 and 3,4-difluorothiophene-2,5-diylbis(trimethylstannane) (M3). The detailed synthetic procedures can be found in the Supplementary Information.

**Figure 1.**
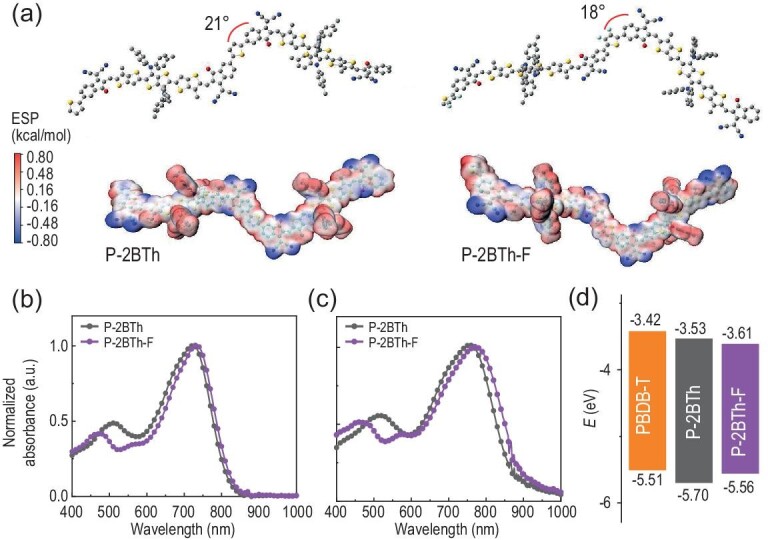
(a) The calculated molecular configurations of **P-2BTh** and **P-2BTh-F**. Absorption spectra of **P-2BTh** and **P-2BTh-F** in dilute chloroform solutions (b) and as thin films (c). (d) Energy levels of PBDB-T, **P-2BTh** and **P-2BTh-F**.

To reveal the molecular conformation difference between **P-2BTh** and **P-2BTh-F**, we adopted the density functional theory (DFT) calculation at the B3LYP/6–31G(d) level to calculate the dihedral angles and surface electrostatic potential. As shown in Fig. 1a, the monomer M1 displays a quasi-planar molecular backbone due to the existence of intramolecular S···O and S···N noncovalent interactions. Then, the dihedral angles between monomer M1/M2 and M1/M3 are 21° and 18°, respectively, indicating that the introduction of fluorine atoms can slightly improve the planarity of molecular backbones. Besides, we calculated the electrostatic potential (ESP) distribution of the polymers by extracting two repeat units for estimation. As shown in Fig. 1a, the fluorinated thiophene in **P-2BTh-F** displays a negative value, indicating a tendency for enhanced intermolecular charge transfer with electron-donating groups. UV-vis absorption spectra of **P-2BTh** and **P-2BTh-F** in dilute solutions and as thin films are shown in Fig. 1b. In dilute solutions, both **P-2BTh** and **P-2BTh-F** display broad absorption in the range of 400–800 nm. Compared with their corresponding solution absorptions, the film absorption spectra of **P-2BTh** and **P-2BTh-F** exhibit a significant red-shift to the range of 400–850 nm. Especially, **P-2BTh-F** with 3,4-difluorothiophene shows a further red-shifted absorption, which can be ascribed to the electron withdrawing effect of the fluorine in **P-2BTh-F**. The wider absorption of **P-2BTh-F** is beneficial for acquiring a higher short-circuit current (*J*_sc_). According to the equation: *E*_g_^opt^ = 1240/*λ*_onset_, the optical bandgaps (*E*_g_^opt^) of **P-2BTh** and **P-2BTh-F** are estimated to be 1.42 and 1.39 eV, respectively. Cyclic voltammetry (CV) measurements are conducted to ascertain the energy levels. Utilizing the equation: *E*_HOMO/LUMO_ = −e(*E*_onset,ox/red_ − *E*_Fc/Fc_ + 4.80), the highest occupied molecular orbitals (HOMOs) and lowest unoccupied molecular orbitals (LUMOs) of **P-2BTh** and **P-2BTh-F** are determined to be −5.70/−3.53 eV and −5.56/−3.61 eV, respectively (Table 1). These findings suggest that the introduction of fluorine atoms can lower the energy levels of the polymers.

**Table 1.** Optical and electrochemical properties of **P-2BTh** and **P-2BTh-F**.

Acceptor	*λ*_max_ (nm)^a^	*λ*_max_ (nm)^b^	HOMO (eV)	LUMO (eV)	*E*_g_^opt^ (eV)
**P-2BTh**	719	756	–5.70	–3.53	1.42
**P-2BTh-F**	730	772	–5.56	–3.61	1.39

a In dilute chloroform solutions.

b As thin films.

**Figure 2.**
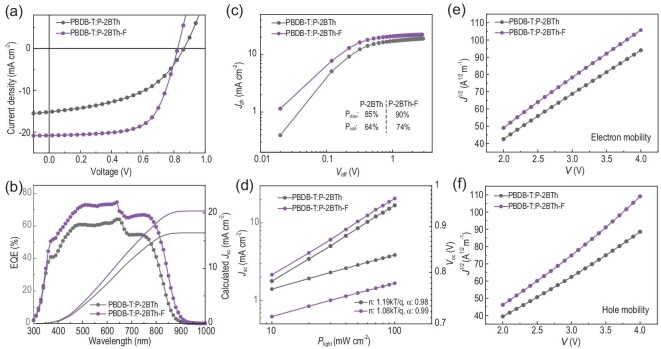
(a) The *J–V* curves; (b) the EQE and integrated *J*_sc_ curves, (c) the *J*_ph_ versus *V*_eff_ curves, (d) dependence of *V*_oc_ and *J*_sc_ on light intensity. (e, f) *J*^1/2^–*V* characteristics of electron and hole-only devices of **P-2BTh-** and **P-2BTh-F**-based blends, respectively, by the space-charge-limited current (SCLC) method.

To investigate the photovoltaic performance of **P-2BTh** and **P-2BTh-F**, conventional devices with a structure of ITO/2PACz/PBDB-T:acceptor (100 nm)/PDINN (10 nm) are fabricated [38]. The devices based on **P-2BTh-F** exhibit an exceptional PCE of 11.06% with an open-circuit voltage (*V*_oc_) of 0.82 V, a short-circuit current (*J*_sc_) of 20.81 mA cm^−2^ and a fill factor (FF) of 64.54% (as shown in Fig. 2 and Table S4). In contrast, **P-2BTh**-based devices demonstrate a lower efficiency of 8.70% with a higher *V*_oc_ of 0.87 V but a lower *J*_sc_ of 16.95 mA cm^−2^ and an FF of 58.38%. In order to characterize the photovoltaic response of OSCs, the external quantum efficiency (EQE) curves of the devices are recorded. As shown in Fig. 2b, **P-2BTh**-based devices exhibit a broad photovoltaic response ranging from 300 to 880 nm, whereas **P-2BTh-F**-based devices demonstrate an even wider photovoltaic response in the range of 300 to 900 nm. Furthermore, **P-2BTh-F**-based devices can achieve EQE values exceeding 70%, significantly higher than those of **P-2BTh**-based devices, which are ∼60%. The broader and higher photovoltaic response of **P-2BTh-F**-based devices accounts for the higher *J*_sc_ values. Furthermore, as shown in Fig. 2c, the *P*_diss_ and *P*_coll_ of **P-2BTh**- and **P-2BTh-F**-based devices, investigated by plotting photocurrent (*J*_ph_) against the effectively applied voltage (*V*_eff_), are as high as 85%/64% and 90%/74%, respectively. Besides, the degree of bimolecular and trap-assisted recombination in devices based on **P-2BTh** and **P-2BTh-F**, as shown in Fig. 2d, can be described by the dependence of *J*_sc_ and *V*_oc_ on the light intensity (*P*_light_) with the formulas of *J*_sc_ ∝ *P*_light_*^α^* and *V*_oc_ ∝ *n*ln*P*_light_, respectively [39]. The *n* and α values of **P-2BTh**- and **P-2BTh-F**-based devices are 1.19 kT/q, 0.98 and 1.08 kT/q, 0.99, respectively, indicating that the trap-assisted recombination in **P-2BTh-F**-based OSCs is suppressed.

The hole/electron mobilities (*μ*_h_/*μ*_e_) of **P-2BTh**- and **P-2BTh-F**-based devices are measured by using the SCLC method with structures of ITO/PEDOT:PSS/polymer: BT**P-**eC9-4F/MoO_3_/Ag and ITO/ZnO/D18: acceptor/PNDIT-F3N/Ag, respectively. The results are shown in Fig. 2e, f and Table S5. The devices based on **P-2BTh** exhibit a *μ*_e_ of 1.80 × 10^−4^ cm^2^ V^−1^ s^−1^, a *μ*_h_ of 1.60 × 10^−4^ cm^2^ V^−1^ s^−1^ with a *μ*_h_/*μ*_e_ ratio of 0.91. However, the *μ*_h_ and *μ*_e_ values of **P-2BTh-F**-based devices are higher and more balanced (*μ*_e_ = 2.25 × 10^−4^ cm^2^ V^−1^ s^−1^, *μ*_h_ = 2.35 × 10^−4^ cm^2^ V^−1^ s^−1^, *μ*_h_/*μ*_e_ = 1.04), which can well explain the superior photovoltaic performance of **P-2BTh-F**-based OSCs.

**Figure 3.**
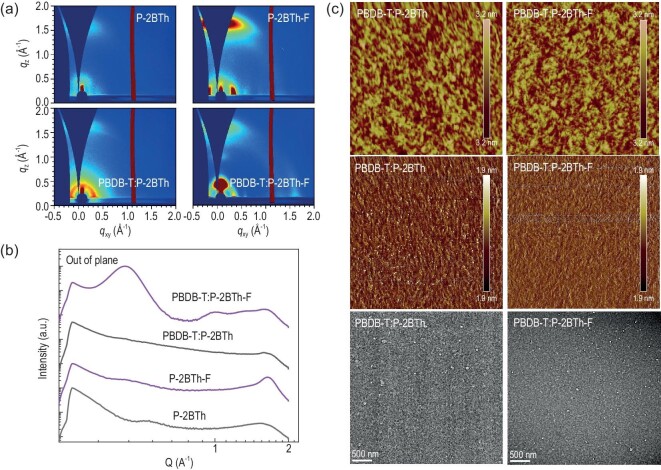
2D-GIWAXS patterns (a) and the 1D profiles along the out-of-plane (OOP) directions (b) of blend films. (c) AFM height and phase images (5 μm × 5 μm) and TEM images of PBDB-T:**P-2BTh** and PBDB-T:**P-2BTh-F** blend films.

The molecular stacking and orientation in the neat and blend films can be analyzed using grazing-incidence wide-angle X-ray scattering (GIWAXS) measurements. As depicted in Fig. 3a, there is a notable difference in the molecular stacking and arrangement of **P-2BTh** and **P-2BTh-F** in their respective neat films. More specifically, **P-2BTh** exhibits a weak face-on molecular orientation with a larger π–π stacking distance of 4.21 Å (1.49 Å^−1^), whereas its fluorinated counterpart **P-2BTh-F** tends to form a strong face-on molecular orientation with a shorter π–π stacking distance of 3.83 Å (1.63 Å^−1^). According to the Scherrer equation, CCL = 2π × 0.89/FWHM (full width at half maxima), the crystal coherence length (CCL) is derived to analyze the molecular stacking in the films of acceptors, and the CCL values of the (010) diffraction in the OOP direction are 14.90 and 32.12 Å for the neat **P-2BTh** and **P-2BTh-F** films, respectively. Therefore, the **P-2BTh-F** film exhibits higher crystalline quality with a shorter π–π stacking distance, which facilitates charge transport in the active layer. As for the blend films, both PBDB-T:**P-2BTh** and PBDB-T:**P-2BTh-F** adopt face-on molecular orientations, as evidenced by 1D profiles and 2D patterns, and exhibit a (010) diffraction peak located at 1.66 Å^−1^, corresponding to a π–π stacking distance of 3.78 Å. Notably, the intensities of both the π–π and the lamellar diffraction peaks in the PBDB-T:**P-2BTh-F** blend film are higher compared to those in the PBDB-T:**P-2BTh** blend film. Based on these results, a higher crystallinity and more ordered packing structure are formed in both the neat and blend films based on **P-2BTh-F**, which is beneficial for charge transport and leads to improved photovoltaic performance.

To gain deeper insights into the influence of morphology on photovoltaic performance, the atomic force microscopy (AFM) and transmission electron microscopy (TEM) measurements were performed (Fig. 3c). According to the AFM measurement, the blend films based on **P-2BTh** and **P-2BTh-F** display root-mean-square (RMS) roughness values of 0.76 and 1.31 nm, respectively. In contrast, the PBDB-T:**P-2BTh-F** blend film exhibits a distinct nanoscale interpenetrating network structure, whereas the PBDB-T:**P-2BTh** blend film shows a slightly larger phase separation without the formation of fiber morphology. The same phenomenon was also observed in the TEM image.

**Figure 4.**
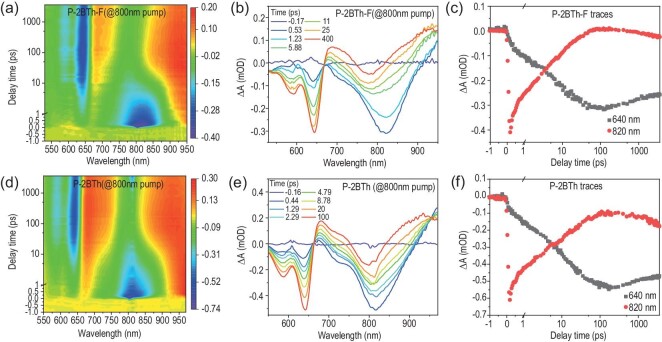
TA results of (a–c) PBDB-T:**P-2BTh** and (d–f) PBDB-T:**P-2BTh-F** blend films pumped at 800 nm. (a, d) Contour plots of the time-resolved absorption difference spectra; (b, e) TA spectra at different delay times; (c, f) kinetic traces at the selected wavelength.

Femtosecond transient absorption (fs-TA) spectra have been recorded for the blend films, allowing us to dive into the intricate hole-transfer dynamics, as depicted in Fig. 4 [40–42]. For the blend films, a low-power pump beam operating at 800 nm is specifically chosen to exclusively excite the acceptors, given the stark contrast in absorption ranges between the polymer donor (PBDB-T) and polymer acceptors (**P-2BTh** and **P-2BTh-F**). As clearly observed in Fig. 4, upon excitation, blend films exhibit strong ground state bleach (GSB) peaks in the longer wavelength range (660–860 nm), accompanied by a gradual increase in negative signals in the shorter wavelength range (530–660 nm). These signatures correspond to the generation of donor excitons and the progressive hole transfer process, respectively. Furthermore, kinetic traces were obtained at specific wavelengths to quantitatively assess the hole-transfer dynamics. As evident in Fig. 4, the attenuation of the GSB signal at 800 nm (indicative of acceptor units in polymers) coincides with an enhancement in the GSB signal at 640 nm (reflective of donor units in polymers). These observations underscore the efficient hole transfer from acceptor units to donor units within the polymer matrix. The rising process of GSB in the short wavelength region directly reflects the hole transfer kinetics. By fitting the GSB rise signal of the donor unit with a double exponential function, the time constants of the blend films can be determined as follows: PBDB-T:**P-2BTh** (*τ*_1_ = 1.92 ± 0.39 ps, *τ*_2_ = 25.20 ± 1.81 ps) and PBDB-T:**P-2BTh-F** (*τ*_1_ = 1.47 ± 0.11 ps, *τ*_2_ = 18.77 ± 0.65 ps), where *τ*_1_ and *τ*_2_ are assigned as the ultrafast exciton dissociation at the interface and the diffusion of the exciton in the domain, respectively. The PBDB-T:**P-2BTh-**based film exhibits fast exciton dissociation at the interface, which is in accordance with the better performance of corresponding devices.

**Figure 5.**
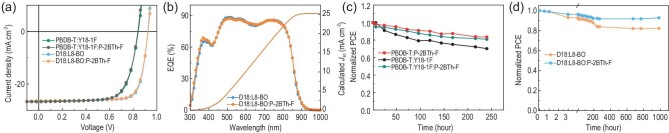
(a, b) *J–V* and EQE of binary and ternary OSCs; (c, d) the stability test of devices under placement conditions.

According to previous literature, OSCs that utilize polymers as acceptor materials typically exhibit superior stability compared to those based on small-molecule acceptors. In this study, we employed **P-2BTh-F** as the third component, incorporated into the PBDB-T:Y18-1F and D18:L8-BO systems, to fabricate ternary OSC devices (Fig. 5 and Table 2). The device structure is ITO/PEDOT:PSS/active layer/PFNDI-F3N/Ag and the photovoltaic performance of the ternary devices is comparable to that of the binary ones. Specifically, D18:L8-BO:**P-2BTh-F**-based devices exhibit an elevated PCE of 19.45% compared to the binary devices. More importantly, the stabilities of the ternary devices are significantly improved after the addition of the polymer acceptor **P-2BTh-F**. These results indicate that **P-2BTh-F** can serve as a potential stabilizer to enhance device stability without compromising its performance.

**Table 2.** Photovoltaic parameters of binary and ternary OSCs.

Devices	*V*_oc_ (V)	*J*_sc_ (mA cm^−2^)	FF (%)	PCE (%)
PBDB-T:Y18-1F	0.84	26.71	72.03	16.19 (16.10)
PBDB-T:Y18-1F:**P-2BTh-F**	0.83	26.78	71.58	16.07 (16.02)
D18:L8-BO	0.92	26.43 (25.26)^a^	78.18	19.31 (18.98)^b^
D18:L8-BO:**P-2BTh-F**	0.92	26.47 (25.30)^a^	79.09	19.45 (19.02)^b^

a Calculated by EQE measurements.

a bAverage PCE of ten devices.

## CONCLUSION

Driven by the quest to fabricate low-cost and stable OSCs, this work focused on designing and synthesizing two innovative polymerized nonfused ring acceptors, namely **P-2BTh** and **P-2BTh-F**. These polymers incorporate thiophene and fluorinated thiophene as the π-bridge linker, respectively. Our findings reveal that **P-2BTh-F** exhibits a more planar molecular backbone, displays a red-shifted absorption spectrum, demonstrates enhanced crystallinity, and possesses superior charge carrier mobility. Furthermore, all-polymer solar cells based on the PBDB-T:**P-2BTh-F** blend achieve a remarkable PCE of 11.06% with exceptional stability. Notably, **P-2BTh-F** can also be utilized as a functional additive in high-performance binary photovoltaic systems, enhancing the stability of devices composed of polymer donors and small-molecule acceptors like PBDB-T:Y18-1F and D18:L8-BO. The resulting ternary devices not only exhibit comparable photovoltaic performance but also possess significantly improved storage stability. All in all, our stability- and cost-oriented strategy demonstrates the potential of **P-2BTh-F** as a promising material for highly efficient and exceptionally stable OSCs. These findings provide valuable theoretical insights for the future commercialization of organic photovoltaic applications.

## Supplementary Material

nwae258_Supplemental_File
