# Supplementary material for: Highly efficient and stable binary and ternary organic solar cells using polymerized nonfused ring electron acceptors
Source: Natl Sci Rev. 2024 Aug 12;11(9):nwae258. doi: 10.1093/nsr/nwae258 (PMC11350609; doi:10.1093/nsr/nwae258)
Supplement: nwae258_Supplemental_File [file nwae258_supplemental_file.docx]

Supporting Information

Highly Efficient and Stable Binary and Ternary Organic Solar Cells Using Polymerized Nonfused Ring Electron Acceptors

Xiaodong Wang ^1^, Nan Wei ^3^, Ya-nan Chen ^1^, Guangliu Ran ^4^, Andong Zhang ^1^, Hao Lu^1,2^*, Zhengdong Wei ^2^, Yahui Liu ^1^*, Wenkai Zhang ^4^, Zhishan Bo ^1,3^*

1. College of Textiles & Clothing, State Key Laboratory of Bio-fibers and Eco-textiles, Qingdao University, Qingdao 266071, China
E-mail: [luhao@qdu.edu.cn](mailto:luhao@qdu.edu.cn), liuyh@qdu.edu.cn, [zsbo@bnu.edu.cn](mailto:zsbo@bnu.edu.cn)
2. College of Materials Science and Engineering, Qingdao University, Qingdao, 266071 P. R. China.

3. Beijing Key Laboratory of Energy Conversion and Storage Materials, College of Chemistry, Beijing Normal University, Beijing, 100875, China.
4. Department of Physics and Applied Optics Beijing Area Major Laboratory, Beijing Normal University, Beijing 100875, China.

**1.1. Material and Instruments**

Unless otherwise noted, all chemicals were commercially purchased and used without further purification. Column chromatography was carried out on silica gel (200-300 mesh). ^1^H and ^13^C NMR spectra were recorded on a Bruker AV 600 spectrometer. UV-visible absorption spectra were obtained on a PerkinElmer UV-vis spectrometer model Lambda 750. Atomic force microscopy (AFM) measurements were performed under ambient conditions using a Digital Instrument Multimode Nanoscope IIIA operating in the tapping mode. The thickness of blend films was determined by a Dektak 6 M surface profilometer. The electrochemical behaviour of the polymers was investigated using cyclic voltammetry with a standard three-electrode electrochemical cell in a 0.1 M Bu_4_NPF_6_ solution in CH_3_CN at room temperature under an atmosphere of nitrogen with a scanning rate of 0.1 V/S. A Pt plate working electrode, a Pt wire counter electrode, and an Ag/AgCl (0.01 M in CH_3_CN) reference electrode were used. The experiments were calibrated with the standard ferrocene/ferrocenium (Fc) redox system and assumption that the energy level of Fc is 4.8 eV below vacuum. GIWAXS measurements were conducted on a Xenocs SAXS/WAXS system with X-ray wavelength of 1.54 Å.

Density functional theory (DFT) calculations were conducted with Gaussian 09 software with a functional of B3LYP and a basis set of 6-311*.^[1]^ To simplify the calculations, methyl and methoxy groups were substituted for alkyl and alkoxy side chains. Electrostatic potential (ESP) analysis was carried using Multiwfn software.^[2]^

**1.2. Fabrication and characterization of solar cells**

Pre-patterned ITO-coated glass substrates were cleaned with detergent and ultrasonicated in deionized water, acetone and isopropanol for 20 min each and subsequently dried in an oven overnight. Device was fabricated with a conventional device structure of ITO/PEDOT:PSS/active layer /PDINN/Ag. PEDOT:PSS (Heraeus Clevios P VP. AI 4083, filtered at 0.45 μm) was spin-cast onto the ITO surface at 4000 rpm for 30 s (the thickness is about 40 nm), and baked at 150 °C for 15 min in air. Concentrations of the PBDB-T and D18 are 5 and 4 mg/mL, respectively. Use the method mentioned in the manuscript to prepare the active layer, with a thickness controlled at 120nm. And the active layers were spin-coated in a N_2_-filled glove box. After the layers were fully dried, the methanol solution of PDINN (1 mg/mL) was spin-coated on the BHJ layer at 3000 rpm. Finally, a 100 nm Ag layer was thermally deposited in vacuum (below 10^-7^ Torr). Photovoltaic cells were fabricated on the substrate with an effective area of 0.04 cm^2^. The *J*-*V* curves were recorded in glove box at approximately 25 °C using an instrument from Enli Technology Ltd., Taiwan (SS-F53A) under AM 1.5G illumination (AAA class solar simulator, with an intensity of 100 mW cm^-2^ calibrated with a standard single crystal Si photovoltaic cell). External quantum efficiency (EQE) measurements were conducted in air without encapsulation. The EQE data were obtained using a solar cell spectral response measurement system (QER3011, Enli Tech-nology Co. Ltd), and the intensity was calibrated with a standard single crystal Si photovoltaic cell.

**1.3. Fabrication and characterization of the hole/electron-only devices**

Devices with the architecture of ITO/PEDOT:PSS/active layer/MoO_3_/Ag and ITO/ZnO/active layer/PDINO/Ag were applied to construct the hole and electron-only device, respectively. The active layers were prepared under the optimal conditions.

According to the Mott-Gurney equation: *J* = 9*εε*_0_*μ*/(8*L*^3^)*V*^2^, the mobility can be described as *μ*=$\frac{{8JL}^{3}}{{9{}_{0}V}^{2}}$. Herein, *ε* denoted the dielectric constant of the blended film based on organic materials, and it was assumed to be constant (3.0); *ε*_0_ = 8.85419×10^-12^ F m^-1^, which meant the permittivity of the vacuum; *μ* represented the zero-field mobility; *J* was the current density; *L* was the thickness of the films; and *V* = *V*_appl_ − *V*_bi_; *V*_appl_ and *V*_bi_ were the applied voltage to the device and the build-in voltage, respectively.

**1.4. Ternary OSCs device preparation**

The ternary OSCs device was fabricated with a conventional device structure of ITO/PEDOT:PSS/active layer /PFNDI-F3N/Ag. Firstly, the PEDOT:PSS (Heraeus Clevios P VP. AI 4083, filtered at 0.45 μm) was spin-coated onto the ITO surface at a rotation speed of 4000 rpm for a duration of 30 seconds, resulting in a uniform layer of approximately 40 nanometers in thickness. Subsequently, this layer was carefully baked at a temperature of 150°C for a period of 15 minutes in air to ensure dryness. Next, the hot active layer solution was spin-coated onto the pre-treated substrate. (Note: For the ternary device, only the mass ratio of the two acceptors was varied; The concentration of PBDB-T was maintained identical to the binary device. In the case of D18, its dissolution concentration was 4 mg/ml, dissolved in CF at 50°C.) After the active layers had thoroughly dried, a methanol solution of PFNDI-F3N (0.5 mg/mL) was spin-coated onto the BHJ layer at 2000 rpm. The subsequent steps were identical to those of the binary device.

**1.5. Device preparation for stability testing**

Device was fabricated with a conventional device structure of ITO/PEDOT:PSS/active layer /PFNDI-F3N/Ag. The preparation method is consistent with 1.2.

Figure S1. CV curves of P-2BTh and P-2BTh-F using ferrocene (Fc) as an internal standard.

Table S1. Device parameters of OSCs

| Acceptor | Ratio | *V*_oc_ (V) | *J*_sc_ (mAcm^-2^) | FF (%) | PCE (%) |
| --- | --- | --- | --- | --- | --- |
|  | 1.2:1 | 0.87 | 15.42 | 49.30 | 6.65 |
| **P-2BTh** | 1:1 | 0.87 | 16.95 | 58.38 | 8.70 |
|  | 1:1.2 | 0.86 | 14.81 | 51.32 | 6.60 |
|  | 1.2:1 | 0.83 | 19.98 | 55.83 | 9.28 |
| **P-2BTh-F** | 1:1 | 0.82 | 20.81 | 64.54 | 11.06 |
|  | 1:1.2 | 0.81 | 18.94 | 54.75 | 8.38 |

Figure S2. TA results of (a) P-2BTh and (b) P-2BTh-F neat films pumped at 800 nm, The TA spectra at selected delay time of (b)P-2BTh and (d)P-2BTh-F neat films.


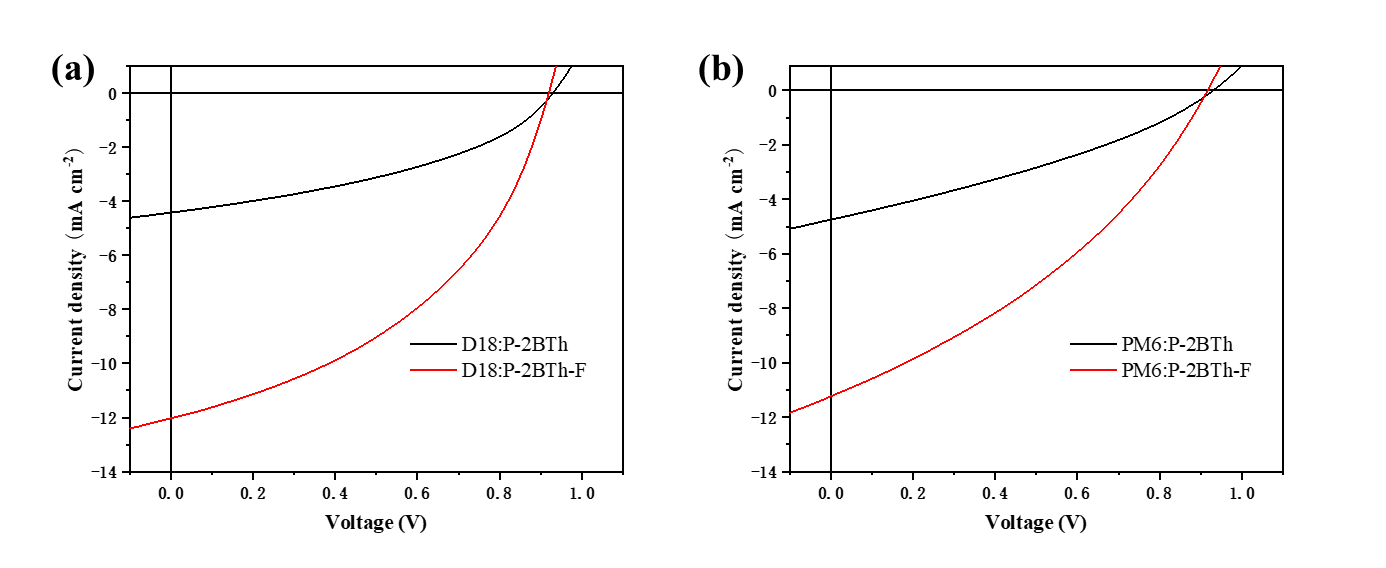


Figure S3. *J-V* curve of OSCs based on PM6 and D18.

Table S2. Photovoltaic data of ternary OSCs.

| D18:L8-BO: P-2BTh-F | *V*oc (V) | *J*sc (mAcm^-2^) | FF (%) | PCE (%) |
| --- | --- | --- | --- | --- |
| 1:1.2:0 | 0.92 | 26.43 | 78.18 | 19.31 |
| 1:1.1:0.1 | 0.92 | 26.47 | 79.09 | 19.45 |
| 1:1:0.2 | 0.91 | 27.01 | 78.51 | 19.22 |

Table S3. Photovoltaic data of OSCs.

| Active layer | *V*oc (V) | *J*sc (mAcm^-2^) | FF (%) | PCE (%) |
| --- | --- | --- | --- | --- |
| D18:P-2BTh | 0.93 | 4.42 | 39.86 | 1.64 |
| D18:P-2BTh-F | 0.92 | 12.03 | 43.10 | 4.78 |
| PM6:P-2BTh | 0.93 | 4.74 | 32.39 | 1.43 |
| PM6:P-2BTh-F | 0.91 | 3.61 | 35.03 | 3.61 |

Table S4. Photovoltaic parameters of OSCs based on **P-2BTh** and **P-2BTh-F**.

| Active layer | *V*_oc_ (V) | *J*_sc_ (mA cm^-2^) | FF (%) | PCE (%) |
| --- | --- | --- | --- | --- |
| **PBDB-T:P-2BTh** | 0.87 | 16.95(15.31) ^a^ | 58.38 | 8.70 (8.40) ^b^ |
| **PBDB-T:P-2BTh-F** | 0.82 | 20.81(20.37) ^a^ | 64.54 | 11.06(10.21)^b^ |

^a^ Calculated by EQE measurements. ^b^ Average PCE of ten devices.

Table S5. The electron and hole mobilities of devices based on **P-2BTh** and **P-2BTh-F**.

| Active layer | *μ*_e_ (cm^2^ V^-1^ s^-1^) | *μ*_h_ (cm^2^ V^-1^ s^-1^) | *μ_h_/μ*_e_ |
| --- | --- | --- | --- |
| PBDB-T:**P-2BTh** | 1.80 x 10^-4^ | 1.60 x 10^-4^ | 0.91 |
| PBDB-T:**P-2BTh-F** | 2.25 x 10^-4^ | 2.35 x 10^-4^ | 1.04 |

**
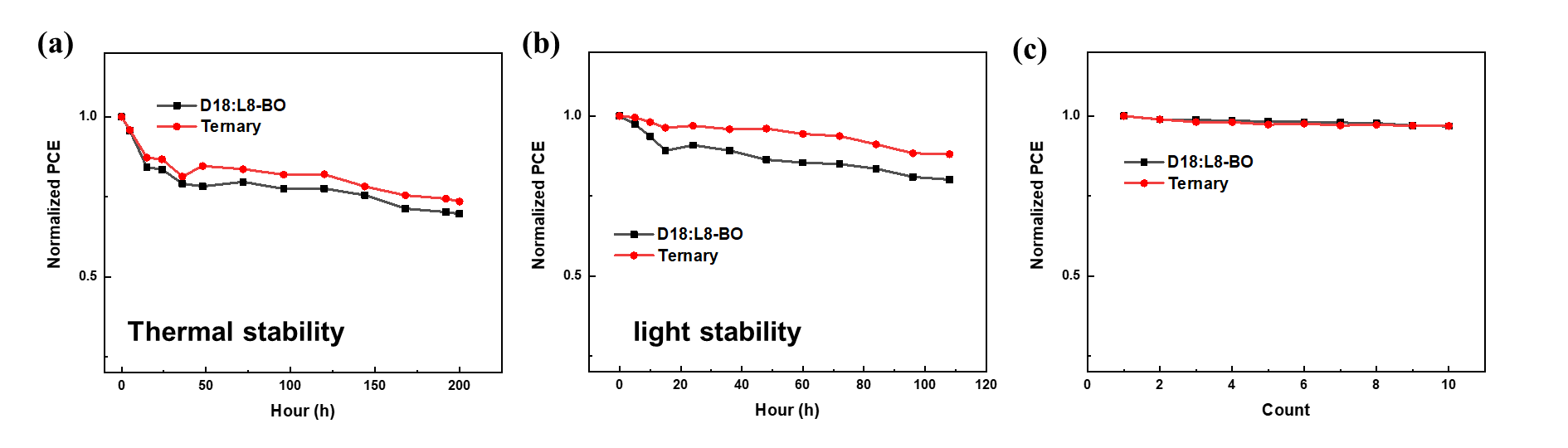
**

Figure S4. The stability test of devices under (a) hotplate (80°C), (b)one sun intensity illumination and continuous multiple tests.


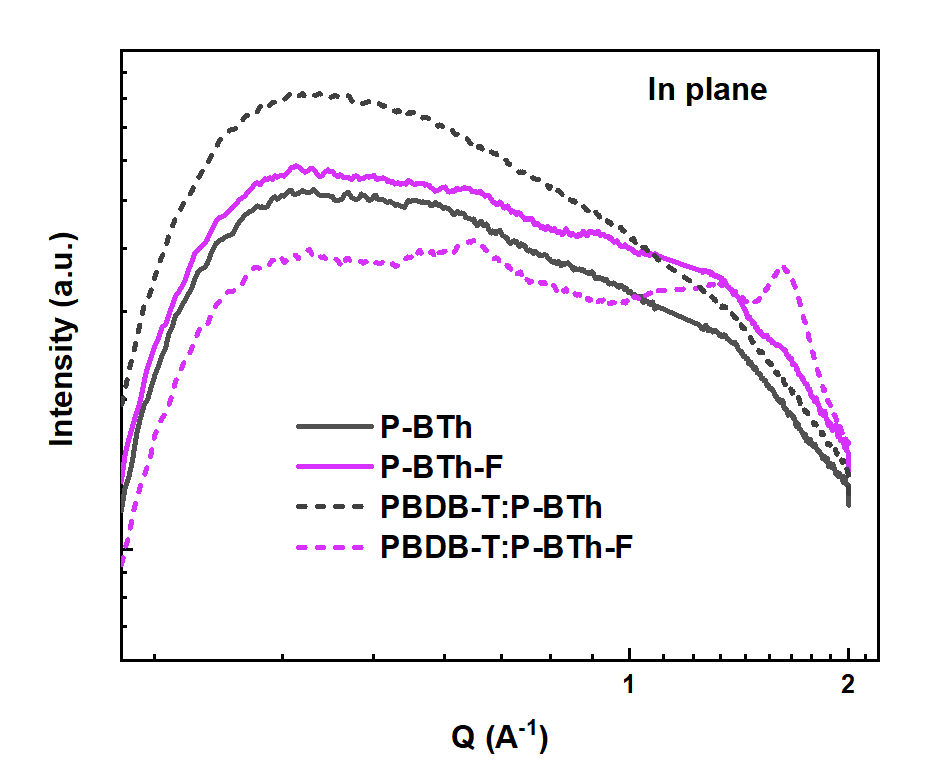


Figure S5. The 1D profiles along the in-plane (IP) directions of pure and blend films.

**Materials and Synthesis.**


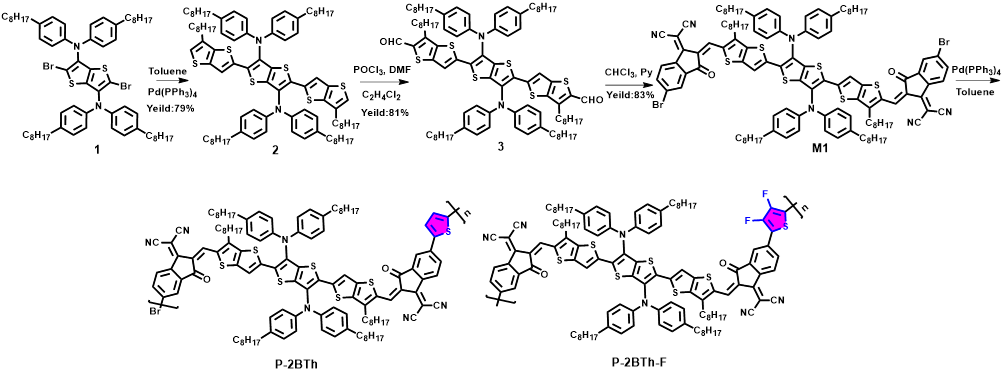


Scheme S1. Preparation of the P-BTh and P-2BTh-F

Compound 1 was synthesized according to previous report (Chin. J. Chem. 2023, 41, 665-671).

Synthesis of compound **2**

Compound **2** can be synthesized by Stille coupling reaction. compound **1** (1000 mg, 0.92 mmol), trimethyl(6-octylthieno[3,2-b]thiophen-2-yl)stannane (1121 mg, 2.7 mmol), Pd(PPh_3_)_4_ (53 mg) were added to toluene under nitrogen. The mixture was reacted at 120 °C for 1 day. After cooling to room temperature, saturated sodium bicarbonate solution was added and extracted with dichloromethane for three times, dried over anhydrous MgSO_4_, filtered and concentrated under reduced pressure. The residue was chromatographically purified on silica gel column eluting with dichloromethane/petroleum ether (3:1, v/v) to give compound **2** (1039 mg, 79%). ^1^H NMR (400 MHz, CDCl_3_) δ: 7.14 (s, 2H), 7.06-7.08 (d, *J*=8Hz, 8H), 7.03-7.05 (d, *J*=8Hz, 8H), 6.84 (s, 2H), 2.58-2.59 (m, 4H), 2.54-2.56 (m, 8H), 1.61-1.63 (m, 4H), 1.54-1.57 (m, 8H)1.25-1.29 (m, 60H), 0.83-0.89 (m, 18H).

Synthesis of compound **3**

Compound **3** can be synthesized by Vilsmeier-Haack reaction. POCl_3_ (0.34 mL, 3.5 mmol) was added to 5 mL DMF under nitrogen at 0 ^o^C. After stirring for 1 h, the formed Vilsmeier reagent was added dropwise to 1,2-dichloroethane (20 mL) solution of compound **2** (500 mg, 0.35 mmol). The mixture was stirred at 0 ^o^C for 30 minutes, and then heated to 85 ^o^C for 24 hours. After cooling to room temperature, saturated sodium bicarbonate solution was added and stirred for 3 hours. The mixture was extracted with dichloromethane for three times; and the combined organic phases were washed with saturated salt solution, dried over anhydrous MgSO_4_, filtered and concentrated under reduced pressure. The residue was chromatographically purified on silica gel column eluting with dichloromethane/petroleum ether (2:1, v/v) to give compound **3** (421 mg, 81%) as an orange solid . ^1^H NMR (600 MHz, CDCl_3_) δ: 9.98 (s, 2H), 7.14 (s, 2H), 7.06-7.08 (d, *J*=8Hz, 8H), 7.03-7.05 (d, *J*=8Hz, 8H), 2.94-2.97 (m, 4H), 2.52-2.54 (m, 8H), 1.64-1.67 (m, 4H), 1.55-1.58 (m, 8H), 1.25-1.29 (m, 60H), 0.85-0.88 (m, 18H). ^13^C NMR (100 MHz, CDCl_3_) δ: 167.85, 152.64, 152.10, 145.04, 144.12, 143.06, 141.82, 138.16, 135.09, 134.28, 131.25, 129.72, 128.74, 122.45, 35.09, 31.96, 31.52, 30.69, 29.87, 29.55, 29.46, 29.42, 29.37, 29.21, 22.72, 14.19.

Synthesis of compound **4**

A mixture of compound **3** (150 mg, 0.13 mmol) and 2-(5-bromo-3-oxo-2,3-dihydro-1H-inden-1-ylidene)malononitrile (169 mg, 0.52 mmol ) in chloroform (20 mL) was carefully degassed and pyridine (0.5 mL) was added. The mixture was stirred and refluxed under nitrogen overnight, and the solvent was removed under reduced pressure. The crude product was purified on silica gel chromatography using dichloromethane/petroleum ether (2:1, v/v) as an eluent to obtain the target compound **4** (167 mg, 83%) as a black solid. ^1^H NMR (400 MHz, CDCl_3_) δ: 8.97 (s, 2H), 8.47-8.49 (d, *J*=8Hz, 2H), 7.97 (s, 2H), 7.75-7.77 (d, *J*=8Hz, 2H), 7.17 (s, 2H), 7.08-7.10 (d, *J*=8Hz, 8H), 7.05-7.07 (d, *J*=8Hz, 8H), 2.95-2.98 (m, 4H), 2.52-2.56 (m, 8H), 1.60-1.63 (m, 4H), 1.53-1.57 (m, 8H), 1.20-1.23 (m, 60H), 0.82-0.87 (m, 18H). ^13^C NMR (100 MHz, CDCl_3_) δ: 186.72, 160.28, 156.27, 152.12, 151.42, 150.77, 145.36, 142.92, 142.13, 139.03, 138.69, 138.44, 136.35, 135.73, 135.67, 134.91, 131.52, 129.41, 126.84, 126.37, 125.56, 122.20, 120.50, 117.06, 68.29, 35.44, 31.97, 31.52, 30.69, 29.63, 29.55, 29.46, 29.44, 29.37, 29.21, 22.71, 14.18.

Synthesis of polymer **P-2BTh**

Under nitrogen protection, compound **4** (150 mg, 0.08 mmol), 2,5-bis(trimethylstannyl)thiophene (30.9 mg, 0.08 mmol), Pd_2_(dba)_3_ (2.2 mg) and P(o-tol)3 (3.6 mg) and degassed toluene were added to 10 mL round bottom flask. After the reaction, the mixture was stirred at 110 °C for 3 h. Then the mixture was poured into methanol (50 mL) and precipitation occurred. Then the resulting mixture was filtered. The obtained crude polymer was dissolved in warm chloroform and then the solution was quickly filtered through a pre-prepared wet silica gel column with hot chloroform (100~200 mesh). The collected chloroform solution was concentrated and precipitated in methanol to get PSMAS as a dark solid (102 mg). GPC: Mn= 5.6 kDa, Mw =14.0 kDa; PDI =2.49.

Synthesis of polymer **P-2BTh-F**

Under nitrogen protection, compound **4** (150 mg, 0.08 mmol), (3,4-difluorothiophene-2,5-diyl)bis(trimethylstannane) (33.4 mg, 0.08 mmol), Pd_2_(dba)_3_ (2.2 mg) and P(o-tol)3 (3.6 mg) and degassed toluene were added to 10 mL round bottom flask. After the reaction, the mixture was stirred at 110 °C for 3 h. Then the mixture was poured into methanol (50 mL) and precipitation occurred. Then the resulting mixture was filtered. The obtained crude polymer was dissolved in warm chloroform and then the solution was quickly filtered through a pre-prepared wet silica gel column with hot chloroform (100~200 mesh). The collected chloroform solution was concentrated and precipitated in methanol to get PSMAS as a dark solid (109 mg). GPC: Mn= 5.8 kDa, Mw =8.6 kDa; PDI =1.48.

[1] G. W. T. M. J. Frisch, H. B. Schlegel, G. E. Scuseria, M. A. Robb, J. R. Cheeseman,, V. B. G. Scalmani, B. Mennucci, G. A. Petersson, H. Nakatsuji, X. Li, M. Caricato, A., J. B. Marenich, B. G. Janesko, R. Gomperts, B. Mennucci, H. P. Hratchian, J. V. Ortiz, A., J. L. S. F. Izmaylov, D. Williams-Young, F. Ding, F. Lipparini, F. Egidi, J. Goings, B., A. P. Peng, T. 1694.991 1693.998 1695.985 1696.998 1691.038 1697.974 1692.040 1693.033 1690.008 1698.997 1699.992 0 1000 2000 3000 4000 Intens. [a.u.] 1690 1692 1694 1696 1698 1700 m/z 34 Henderson, D. Ranasinghe, V. G. Zakrzewski, J. Gao, N. Rega, G. Zheng,, M. H. W. Liang, M. Ehara, K. Toyota, R. Fukuda, J. Hasegawa, M. Ishida, T. Nakajima, Y., O. K. Honda, H. Nakai, T. Vreven, K. Throssell, J. A. Montgomery Jr., J. E. Peralta, F., M. B. Ogliaro, J. J. Heyd, E. Brothers, K. N. Kudin, V. N. Staroverov, T. Keith, R., J. N. Kobayashi, K. Raghavachari, A. Rendell, J. C. Burant, S. S. Iyengar, J. Tomasi, M., J. M. M. Cossi, M. Klene, C. Adamo, R. Cammi, J. W. Ochterski, R. L. Martin, K., O. F. Morokuma, J. B. Foresman, D. J. Fox, Gaussian 09, Gaussian, Inc., Wallingford, CT. 2009.

[2] J. Zhang, T. Lu, PCCP. 2021, 23, 20323
